# Pathogens and Immigrants: A Critical Appraisal of the Behavioral Immune System as an Explanation of Prejudice Against Ethnic Outgroups

**DOI:** 10.3389/fpsyg.2019.02412

**Published:** 2019-10-25

**Authors:** Isabel Kusche, Jessica L. Barker

**Affiliations:** ^1^Aarhus Institute of Advanced Studies, Aarhus University, Aarhus, Denmark; ^2^Interacting Minds Centre, Aarhus University, Aarhus, Denmark

**Keywords:** behavioral immune system, pathogen threat, disgust, contagion avoidance, ethnic outgroups, prejudice, xenophobia, evolutionary psychology

## Abstract

The last two decades have seen the development of a body of literature in evolutionary psychology that seeks to attribute negative attitudes to ethnic and racial minorities and other outgroups to an evolved behavioral immune system (BIS). It hypothesizes that disgust sensitivity, which evolved as protection against pathogen threats, also triggers reactions to cues that are not viscerally disgusting, such as people with unfamiliar features, and thus can explain prejudice toward members of these groups. Such an explanation seems to limit the influence of education, public policy, and rhetoric on those attitudes. Our conceptual analysis shows that this is not the case. Existing hypotheses about why the BIS would be triggered even in the absence of visceral disgust elicitors suggest that general unfamiliarity or atypicality act as cues for this hypersensitive threat detection system. This implies that the impact of the BIS must depend on the cultural and societal context in which people learn not only what is disgusting but also what is typical. The social context of personal interaction with mass media representation of and political debate about immigrants consequently needs to be considered as a decisive factor for any effect of the BIS on attitudes and behavior toward ethnic and racial outgroups. The BIS is therefore not a separate or even superordinate explanation of prejudice, compared to those coming from the social sciences. We conclude that it can offer valuable insights into processes of stigmatization and prejudice, once the role of social learning in the developmental unfolding and activation of psychological mechanisms is taken seriously.

## Introduction

Why do people have a propensity to exclude or otherwise discriminate against certain groups of other people? Evolutionary approaches to stigmatization assume that the tendency to exclude individuals with certain characteristics results from psychological mechanisms that evolved by natural selection because they solved specific problems faced by our human ancestors (Kurzban and Leary, 2001). The last two decades have seen the development of a body of literature in evolutionary psychology that attributes many present-day political attitudes, such as social or political conservatism (Terrizzi et al., 2013), ethnocentrism (Navarrete and Fessler, 2006), and xenophobia (Faulkner et al., 2004), to evolved threat-management mechanisms (Neuberg and Schaller, 2016). Much of this research has focused on the behavioral immune system (BIS), which is hypothesized to produce automatic disgust reactions as an evolved response to the threat of pathogens (Schaller, 2006; Terrizzi et al., 2013). The BIS is considered an example of how the hypersensitivity of threat detection mechanisms can produce prejudices against people who do not actually pose a threat.

Social psychology and sociology have mostly looked in other directions to explain stigmatization, although more recently Phelan et al. (2008) have recognized evolutionary explanations as a promising route for understanding stigma associated with non-infectious diseases and disabilities. Mostly, contributions from these disciplines have pointed to uncertainty about the right way to interact with people who for various reasons look or behave differently from what is considered to be normal (Goffman, 1963), or dominant cultural beliefs that lead to negative stereotyping and othering of certain groups (Link and Phelan, 2001, pp. 368–370). Power differentials and efforts to preserve them have often been identified as the driving force behind stigmatization and prejudice (Elias and Scotson, 1965; Link and Phelan, 2014; Tyler and Slater, 2018).

The BIS approach to negative attitudes toward ethnic outgroups and immigrants superficially ties in with sociological research on dehumanization and disgust-eliciting language in public discourse (Esses et al., 2013; Utych, 2018). The observation that the implicit or explicit equation of immigrants and vermin in public discourse contributes to their rejection resonates with the BIS literature’s focus on associations between parasite stress or pathogen threat and ingroup assortative sociality (Fincher and Thornhill, 2012), anti-immigrant attitudes (Aarøe et al., 2017), and xenophobia (Faulkner et al., 2004). However, different from sociological perspectives, the BIS approach investigates why cues that are not viscerally disgusting traits produce reactions of disgust. It does not try to explain why some people may *consciously* express disgust toward other ethnic groups (Hodson et al., 2013). Instead, the focus is on *unconscious* disgust responses. Higher individual disgust sensitivity, which evolved by natural selection as protection against pathogen threats, is hypothesized to lead to more negative attitudes toward strangers and immigrants even in the absence of consciously detected cues of such threats.

With a growing interest from political science in this line of research, policy implications of its findings are increasingly discussed. The reference to humans’ evolutionary past as the ultimate explanation for some contemporary attitudes and behaviors depicts those not only as unintentional but also as largely outside conscious reflection. Such an explanation can also be misunderstood to mean a limited influence of education, public policy, and rhetoric on those attitudes and behaviors. For example, Kam and Estes (2016) suggest that disgust sensitivity can explain individuals’ policy opinions and preferences and thus influence the demand for protective policies in such diverse policy fields as food safety and immigration, i.e., “policies that most overtly lend themselves to concerns about bodily and societal contamination” (Kam and Estes, 2016, p. 493). Clifford and Jerit (2018), after finding that disgust reactions impair memory and information-seeking of crucial facts related to a perceived threat (in their studies an infectious disease), suggest a possible generalization to public reactions to stigmatized social groups, such as immigrants and ethnic outgroups: “[D]isgust may be an obstacle to perspective taking and learning about other groups/cultures – two common methods for countering prejudice and intolerance” (Clifford and Jerit, 2018, p. 277). An article focusing on explaining anti-immigrant attitudes makes the similar but stronger claim that “the evolved features of the BIS fundamentally change the politics of ethnic inclusivity and frustrate the integrationist route to tolerance as multiculturalism increases in the Western world” (Aarøe et al., 2017, p. 290).

In this article, we show that such a conclusion is not justified, even if one accepts both the theoretical assumptions and the empirical findings of a link between disgust sensitivity and attitudes toward immigrants. This is because the existing literature has not sufficiently considered the interplay between evolved psychological mechanisms and the present social world in which these mechanisms affect behavior. As a result, research linking BIS or disgust to attitudes toward immigrants and other outgroups suffers from two conceptual shortcomings, although to varying degrees. Some take social context into account, but only in the form of individual-level variables such as education, income, or political ideology (e.g., Aarøe et al., 2017). They thus treat the potential role of societal-level factors such as political rhetoric and media coverage in shaping disgust reactions as somewhat irrelevant. Others take media coverage and the emotions it evokes as their starting point (Clifford and Jerit, 2018) and emphasize that many elicitors of disgust are socially constructed (Kam and Estes, 2016). At the same time, however, they treat the psychological mechanism that underlies disgust reactions as a black box and do not consider the possibility of non-visceral triggers of disgust that the BIS concept includes.

By placing the concept of the BIS in the context of arguments from both evolutionary psychology and sociology, we show that its contribution to understanding prejudice against ethnic outgroups is not a separate or even superordinate explanation to those focusing on social context. On the contrary, any effect of the BIS on attitudes and behavior toward ethnic and racial outgroups is mediated by the social context of personal interaction with mass media representation of and political debate about immigrants. For this reason, an evolved BIS is no fundamental obstacle to policies that stress efforts at integration and social learning of tolerance toward unfamiliar people, such as those based on intergroup contact (e.g., Pettigrew and Tropp, 2006). However, in conjunction with social context, the concept can offer a deeper understanding of the circumstances under which prejudice and xenophobia are reproduced.

In section “Disgust and the Behavioral Immune System,” we describe how the BIS concept of disgust relates to and differs from social-psychological and social-anthropological approaches to the role of disgust in shaping attitudes toward ethnic outgroups and immigrants. In section “Ethnic Outgroups and Cues of Pathogen Threat,” we take a closer look at the hypotheses about the BIS and identify a theoretical ambiguity regarding its presumed response to cues of outgroup membership, which can only be resolved by a recourse to social context. We subsequently show in section “Bringing Back the Social Context” that any effect that the BIS may have on attitudes toward ethnic outgroups and immigrants is deeply entwined with the impact of media and political discourse and therefore not a separate or even superordinate explanation for negative attitudes. We conclude that the concept of the BIS can offer valuable insights into how processes of stigmatization and prejudice can be influenced by a psychological mechanism that elicits disgust reactions. It is however essential that it is embedded in a discussion of the contemporary social and political factors that create the context in which that mechanism is triggered.

## Disgust and the Behavioral Immune System

The BIS is considered to be a suite of emotions, cognitions, and behavior that acts reactively and proactively to protect humans from pathogens (Schaller, 2006). It responds to potential cues of disease with the emotion of disgust and thereby triggers avoidance and/or protective behavior in order to reduce the likelihood of contagion or contamination from close contact. Due to the potentially high costs of pathogen contamination, and error in mapping pathogen cues onto actual pathogen threats (e.g., because of a latency between infection and the presentation of symptoms, or because cues are ambiguous), the BIS is expected to be hypersensitive and generate false positive reactions, which means that it can be triggered by cues of pathogens even if there is no actual pathogen threat (Nesse, 2005).

Briefly, a feature of threat detection mechanisms in general (from the BIS and our physiological immune system to animals’ alarm calls in response to predators) is that they are selected to produce “false positives”: this is because the cost of mistakenly responding to a potential threat is less than the cost of ignoring it (error management theory: Haselton and Buss, 2000; Haselton and Nettle, 2006). While these apparent errors are ultimately adaptive, with modern hygiene, many potentially threatening situations do not pose a pathogen risk. Just as the physiological immune system can overreact to generally harmless stimuli (as in food allergies), the first hypothesized step in the false-positive BIS disgust reaction is to misclassify an unfamiliar person or other stimulus as a pathogen threat. The strength of reaction varies across individuals, who consequently can be more or less disgust-sensitive (Tybur et al., 2018).

The concept of the BIS is one important result of a surge in research interest in the emotion of disgust that took off in the late 1990s (Rozin et al., 2016). Its focus on pathogen avoidance as an evolved function of disgust distinguishes it from other attempts to understand the evolutionary origins and social functions of disgust. Although there is wide agreement that pathogen avoidance initially played a fundamental role in the evolution of disgust, there are different views regarding its relevance for understanding the scope of disgust elicitors in contemporary humans.

Most conceptualizations of disgust agree that its evolutionary origin is (partly or entirely) an adaptation to avoid pathogens contained in contaminated food (Rozin et al., 2016). There is less unanimity regarding the question of how to categorize a range of other disgust elicitors within an evolutionary framework. The first influential scale for measuring disgust (Haidt et al., 1994) distinguished seven domains of disgust (food, animals, body products, sex, envelope violations, death, and hygiene) as well as a cross-cutting domain of magical thinking that triggers disgust reactions based on visual similarity with or imagined contagion from disgusting items. A revised disgust scale (Olatunji et al., 2007), based on a psychometric analysis of the initial one, reduced the domains of disgust to three, by merging the disgust elicitors food, animals, and body products into a domain of core disgust, reconceptualizing disgust of death and envelope violation as animal-reminder disgust, and introducing contamination as a separate domain, related to hygiene and aspects previously understood as magical thinking. Another three-domain framework (Tybur et al., 2009) reinterpreted core disgust as pathogen disgust and added sexual and moral disgust as domains in which disgust reactions solve distinct adaptive problems, namely avoiding substances that could cause diseases, avoiding sexual partners and behaviors with a negative impact on long-term fitness, and avoiding individuals with anti-social behaviors that endanger one’s social group.

In particular, the notion of moral disgust and its psychological correlate are controversial. One argument is that verbal expressions of disgust as a reaction to someone’s actions or character are not more than a metaphor (Nabi, 2002) and facial expressions of disgust just signaling devices, communicating reproach and a wish for social distancing (Royzman and Kurzban, 2011; Tybur et al., 2013). Another argument forges a link with cultural anthropology (Douglas, 1966) and conceives moral disgust as a reaction to the threat of spiritual pollution, i.e., a particular type of moral transgression, and thus as a culturally evolved abstraction from non-moral disgust of potentially contaminating substances (Rozin et al., 1999, 2016). Hutcherson and Gross (2011) suggest that there is considerable overlap between disgust, anger, and contempt as negative social emotions related to judgments of others’ attitudes and actions; yet, they find disgust to be most strongly felt in response to transgressions that affect others (as opposed to self) and are not due to incompetence but violate the ethic of community.

The BIS literature, though certainly aware of the conceptual complexities involved in understanding the emotion of disgust (e.g., Lieberman et al., 2012; Clark and Fessler, 2014), by definition focuses on the adaptive function of pathogen avoidance. It looks for and interprets associations between disgust and particular attitudes or behaviors based on the hypothesis that such findings will be related to the function of disgust in avoiding pathogens.

When it comes to linking disgust and attitudes toward ethnic outgroups and immigrants, social science research suggests two ways to make the connection. On the one hand, a politics of disgust can use stereotypical characterizations of certain groups to elicit the emotion of disgust in processes of “othering” (Douglas, 1966; Tyler, 2013; Round and Kuznetsova, 2016). The BIS could be understood as the psychological basis that ensures the effectiveness of such a politics. On the other hand, people perceive immigrants as posing different threats, such as economic, cultural, or security threats (Stephan and Stephan, 2000; McLaren and Johnson, 2007; Hellwig and Sinno, 2017) – a list that could be complemented by adding pathogen threat.

The BIS literature however follows a different route. It seeks a link that precedes public discourse and the labeling of certain groups as pathogen threats. Although not incompatible with the notion of explicit stereotypes about dirtiness and revolting behavior triggering disgust reactions (Clifford and Piston, 2017), the BIS approach focuses on unconscious, non-discursive cues. The assumption is that the BIS uses cues from the environment that are correlated with pathogen risk or were correlated with it in the environment in which this psychological mechanism was selected. We now turn to the arguments that ethnic outgroups are one such cue to which a hypersensitive BIS might react.

## Ethnic Outgroups and Cues of Pathogen Threat

If the BIS can be triggered by ethnic outgroups, it must categorize them as posing a risk of infection. Several hypotheses are given for a psychological mechanism that has evolved to react to cues of general unfamiliar appearance which ethnic outgroups present. The first hypothesis is that contact with previously unknown groups could increase the risk of exposure to novel pathogens. Since such pathogens have not co-evolved with the local population, locals’ physical immune system lacks defenses against them (Thornhill and Fincher, 2014). Consequently, avoidance of even healthy-looking strangers could have provided fitness benefits for our ancestors (Faulkner et al., 2004; Navarrete and Fessler, 2006). This would have made foreigners or strangers a relevant category for our ancestors’ survival and reproduction, which is why the human mind would have evolved an automatic disgust reaction to unfamiliar-looking outgroups.

The notion that the BIS reacts to cues of outgroup membership *per se* (rather than unfamiliarity, of which outgroup membership is one aspect) has met criticism. First, it is questioned whether pathogens carried by outgroups are generally more dangerous to locals than co-evolved pathogens (De Barra and Curtis, 2012). Second, ancestral humans did not travel large enough distances to encounter outgroups with pathogens radically different from their own (van Leeuwen and Petersen, 2018). Third, avoiding individuals from outgroups would not have protected against novel pathogens if some ingroup individuals interacted with members of an outgroup, which was likely due to other fitness benefits that could be gained from such interaction (Robinson and Barker, 2017); infectious pathogens originating in the outgroup would still have spread in the ingroup, but those avoiding outgroup contact would have forgone the benefits from it (van Leeuwen and Petersen, 2018). Empirical studies also raise doubt about the BIS directly reacting to outgroup membership (Tybur et al., 2016; Petersen, 2017).

A second, complementary hypothesis is that the BIS reacts to outgroups because individuals from such groups could be more likely to behave in ways that violate local customs implicitly relevant for avoiding diseases from local parasites, such as rules regarding hygiene or food preparation (Faulkner et al., 2004; Tybur et al., 2016). Such rules could even be considered a part of the BIS, since their (cultural) evolution once brought fitness benefits by neutralizing pathogen threats (Thornhill and Fincher, 2014). Thus, a hypersensitive BIS could take cultural unfamiliarity (rather than outgroup membership itself) as a cue of potential rule violation, triggering a disgust reaction (Aarøe et al., 2017).

A third hypothesis is that the BIS reacts to physical unfamiliarity not because it is a cue for outgroup membership *per se* but because physically unfamiliar traits resemble cues that were once correlated with disease. A hypersensitive BIS might react to general unfamiliarity, including cues of foreignness, because it mistakes atypical appearance for physical features that infected people may display (Aarøe et al., 2017; Petersen, 2017).

To what extent could atypical physical characteristics (parts of the body or bodily movements) be correlated with disease? This can be seen as a continuum, ranging from direct signs of disease, such as sores or swellings (Oaten et al., 2011; Lieberman et al., 2012), on one end, to a less well-defined other end with traits that were or are at least weakly correlated with pathogen threats. For example, some bodily features of obesity resemble symptoms of infectious conditions likely to have been present in ancestral environments (Park et al., 2007).

When it comes to ethnic differences, general physical unfamiliarity is implied to be a cue weakly correlated with disease threats. But the relevant aspects of unfamiliarity are often not specified. Aarøe et al. (2017), p. 278) offer the example of skin color to illustrate how immigrants may be physically unfamiliar in a way relevant to the BIS. As some diseases do indeed affect the color and appearance of human skin, this example illustrates a specific trait that the BIS could classify as relevant. Other ways in which physical unfamiliarity of ethnic outgroups might trigger the BIS are an open question that is ripe for future research.

To whatever understanding of unfamiliarity one subscribes, it is crucial to note that the assessment of whether a trait is typical or atypical, and how well it is correlated with disease, presupposes previous learning. Distinguishing between what is familiar and unfamiliar requires knowledge about what certain physical traits, e.g., faces or body morphology, as well as certain activities, e.g., preparing or eating food, typically look like. Cues of unfamiliarity may also involve the cognition and appraisal of learned labels related to disease (Oaten et al., 2011). In the context of immigration, such knowledge may include stereotypes about whether immigrants live in hygienic conditions (Tsuda, 1998, p. 344) or about the prevalence of infectious diseases in countries from which immigrants typically come. As a psychological mechanism to assess pathogen risk, the BIS would process such stereotypes as disease cues just as it would process physical traits. But stereotypes are cues that are learned in social contexts, in which they may serve many functions that have nothing to do with protection against disease, for example the justification of power differentials (Phelan et al., 2008).

Against this social background, it would seem untenable to conceive the impact of the BIS as independent of the cultural and broader societal context in which people learn what is typical and what is disgusting. Yet the research that considers the link between the BIS or disgust and negative attitudes to ethnic outgroups or immigrants has generally overlooked this fundamental relationship between social context and disgust reaction. It either takes social context into account only as individual-level variables; or it acknowledges the role that social context in the form of political rhetoric and mass media plays as a potential source of disgust elicitors, but only considers explicit elicitors of disgust and not the effect on perceptions of familiarity and typicality to which the BIS may react.

Early examples of the focus on individual-level variables are the studies reported in Faulkner et al. (2004). They test the hypothesis that disease-avoidance mechanisms (the concept of a BIS was not established yet in the field) influence xenophobic attitudes. They examine effects of chronic and temporary perceptions of vulnerability to disease on reactions to subjectively foreign people, based partly on correlational findings and partly on experimental manipulation. The results indicate that a higher perceived vulnerability to disease amplifies xenophobic attitudes, not as a result of a rational risk assessment but of an automatic heuristic. The article stresses that subjective perceptions of familiarity and foreignness seem to be fundamental for the effect and that both individual and situational differences in perceived vulnerability to disease correspond to differences in xenophobic attitudes. Yet the studies do not address the social context in which perceptions of familiarity and foreignness are shaped.

This is of course perfectly justified in terms of a division of labor in research. However, it becomes an issue once the question of policy implications is posed and/or, by leaving out the social and cultural context, a psychological disease-avoidance mechanism is treated as a separate or even competing explanation. An example is Aarøe et al. (2017), who explore the relationship between the sensitivity of the BIS and attitudes toward immigration in the US and Denmark, employing self-report and physiological measures in large-scale surveys and laboratory experiments. The authors find that greater sensitivity of the BIS is associated with greater opposition to immigration, and that cues of disease protection, as well as cues of the physical and cultural familiarity of the immigrant, weaken that relationship. They also find that greater disgust sensitivity is associated with lower approval of situations that involve contact with immigrants who already live in a community, and that cues about the immigrants’ willingness and effort to integrate do not affect this relationship. With these findings, the authors claim that the BIS poses a fundamental obstacle to policies that stress efforts at integration and social learning of tolerance toward unfamiliar people.

The theoretical premise of this work and its suggested policy implications is that the hypersensitive BIS reacts to cues of physical and cultural unfamiliarity, which leads to false-positive identification of pathogen threats, resulting in an unconscious disgust reaction that leads in turn to avoidance and rejection of the people perceived to carry the threat. The only suggestion that this unconscious mechanism is mediated by social context can be found in an online appendix making the important qualification that “substantial and continuous personal contact with immigrants living in the society” (Aarøe et al., 2017, online appendix A12) could have the effect of making people more familiar with ethnic and racial differences.

**Figure 1:**
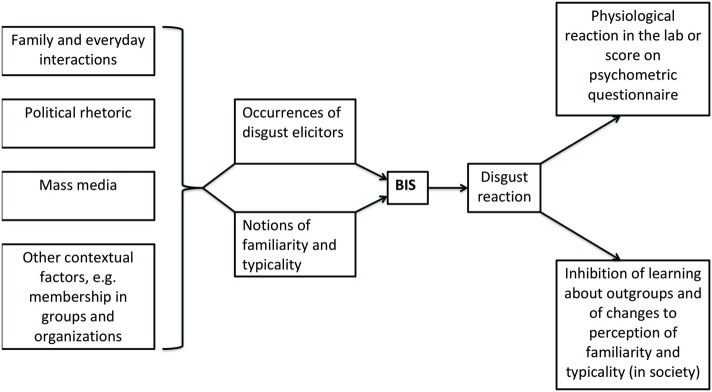
Impact of societal factors on the BIS and (societal) effects of triggered disgust reactions. Individuals are exposed to and learn about disgust elicitors as well as familiar/typical appearance and behavior in social contexts, including family, daily interactions, politics, and mass media. The triggering of an individual’s BIS is influenced by both explicit disgust elicitors and notions of familiarity/typicality. This reaction can be quantified by physiological or psychometric measures. Such data imply that the disgust reaction may prevent learning about outgroups and consequently inhibit changes to perceptions of familiarity and typicality in society.

On the other hand, contributions that have a broader notion of relevant social context and consider mass media or political statements tend not to conceive this context as influencing perceptions of familiarity but only as a potential source of disgust elicitors. For example, Clifford and Jerit (2018) characterize the relationship between disgust reactions and learning as one in which a triggering of the former inhibits the latter. Different from Aarøe et al. (2017), they focus on the role of mass media coverage in presenting disgust-eliciting triggers. Yet, also different from Aarøe et al. (2017), they do not discuss the psychological mechanism that underlies the disgust reaction and the possibility of non-visceral cues as triggers. As a result, the role of learning in connection to disgust needs a more comprehensive reflection: on the one hand, unconscious disgust may be an obstacle toward learning (about other groups and in general); on the other hand, the psychological mechanism that is responsible for disgust reactions operates in a social context in which learning about the attributes to which the BIS may react, namely familiarity and typicality, is always already taking place (Figure 1).

## Bringing Back the Social Context

Mass media and the broader social context beyond personal contact play an important role when it comes to perceiving immigrants as familiar or unfamiliar, both physically and culturally. A lack of encounters with immigrants in a relatively homogenous society such as Denmark or a highly segregated society such as the U.S. (Aarøe et al., 2017) is mostly not the result of active avoidance but of their frequent absence in the everyday life of the majority in the first place. However, such settings do not lack exposure to immigration, because that happens to a large degree *via* mass media. Decisions to include or exclude minorities in contexts unrelated to immigration, especially on television, will consequently influence whether they are perceived as unfamiliar. When immigrants *are* depicted in the mass media, their portrayal can either highlight cultural unfamiliarity or contribute to making the unfamiliar familiar by emphasizing commonalities. The existence of such effects is suggested by research on children’s television and prejudice reduction (Graves, 1999) and the differential effect of public versus private television consumption on anti-immigrant sentiments (Jacobs et al., 2017).

Mass media and the often stereotypical depiction of immigrants are a key part of the social context in which individuals form attitudes about those immigrants (Esses et al., 2013). These depictions are also influenced by the political debate about immigration. The current political discourse in many countries contains various cues that, based on the logic of the BIS, make favorable attitudes toward immigrants less likely. It tends toward emphasizing difference, in particular religious differences between Islam and Christianity (Yilmaz, 2012). But it may also enhance cues of cultural unfamiliarity in other, more indirect ways. If the original fitness benefits of responding to this type of cue derived from an avoidance of behaviors that violated local norms, some of which had an effect on the likelihood of exposure to pathogens (Murray and Schaller, 2012; Tybur et al., 2016), the depiction of immigrants as norm-violators would foster their perception by the BIS as culturally unfamiliar.

Political discourse has subscribed to such depictions in various forms. It has fed a stereotype according to which immigrants tend to be involved in crime or terrorism (Ceobanu, 2011; Sohoni and Sohoni, 2014). It has focused on the distinction between legal and illegal immigrants, which means that immigrants have regularly been depicted as violating laws about entering and living in a country. Political discourse has also increasingly addressed immigration as a problem in the context of welfare state redistribution, with immigrants being framed as less deserving of welfare benefits than natives (Aarøe and Petersen, 2014; Jorgensen and Thomsen, 2016) or as welfare abusers (Brown, 2016). This is based on an implicit norm of reciprocity, which immigrants violate when they claim benefits without having contributed to the welfare state that grants them.

The relationship between the immigration debate and BIS responses is thus two-way, whereas existing research (Kam and Estes, 2016; Aarøe et al., 2017) only considers how BIS responses might influence the immigration debate. For example, in a vignette experiment and survey with U.S. participants, Aarøe et al. (2017) investigate the correlation between disgust sensitivity and opposition to immigration, and find that it is weakened by cues about the immigrants’ physical and cultural familiarity but not by cues about their willingness and effort to integrate. The finding seems to support the BIS hypothesis, since motivation to integrate does not reduce an unconsciously perceived pathogen threat. The authors highlight the motivation to integrate as the main route to increasing tolerance that research has identified. They therefore claim that they are “demonstrating how the behavioral immune system can undermine established pathways to ethnic tolerance in political science research” (Aarøe et al., 2017, pp. 285–286). But they fail to consider that the actual immigration debates to which the participants in their studies have likely been exposed – often emphasizing difference instead of willingness to integrate – may have shaped the reactions of the BIS that they measure in a decisive way.

It is also important to recognize that the geographical origin of an immigrant may act as a cue not only in terms of physical and cultural familiarity, but also in terms of stereotypes about specific regions and their inhabitants (Brader et al., 2008; Hellwig and Sinno, 2017). Once we take the content of the current immigration debate more fully into account, it is clear that it is the debate itself that is an obstacle to more positive attitudes to immigrants, since it tends to depict immigrants as untrustworthy norm violators, i.e., culturally unfamiliar. The BIS approach plays a valuable role in elucidating the psychological mechanism that reacts negatively to such a depiction. But if our evolutionary past gave rise to a BIS sensitive to cultural unfamiliarity as a cue for possible norm violations, it is still our changeable present in which attitudes and behaviors are influenced by explicitly negative depictions of certain groups.

Many empirical studies that have been employed to show the influence of the BIS and pathogen avoidance on various political attitudes (Faulkner et al., 2004; Navarrete and Fessler, 2006; Aarøe et al., 2017) can actually be interpreted in a way that stresses this changeability. These studies typically manipulate cues about disease protection or disease threat to demonstrate their effect on political attitudes. But what they present as support for the claim that pathogen avoidance is a causal factor in the formation of attitudes toward ethnic and racial outgroups can also be read as impressive evidence for the evolved capacity for flexibility of the BIS under changeable environmental conditions. For example, in avoiding contact with others, there is a tradeoff between protecting oneself from pathogens and forgoing social interactions. At times pathogen avoidance may take precedence, whereas under different circumstances the beneficial opportunities of new interactions have more weight (Aarøe et al., 2017, 284). This flexibility, and the ease with which even weak environmental factors – in studies with vignettes, typically verbal cues or hand-washing – alter the influence of the BIS on political attitudes (e.g., Navarrete and Fessler, 2006. pp. 277–278; Huang et al., 2011; Aarøe et al., 2017, p. 285), suggest that we have no reason to assume that the BIS will inevitably come in the way of policies aimed at fostering integration and tolerance. Rather, the behavior resulting from the BIS is highly variable, and depends on the immediately present cues.

## Conclusion

Far from being an obstacle to “the integrationist route to tolerance” (Aarøe et al., 2017, p. 290), the role of the BIS in attitudes toward immigrants and ethnic outgroups actually highlights both the necessity and feasibility of fostering tolerance, provided that peaceful interaction is the policy goal and not the stigmatization of outgroups for political gain. Familiarity with physical and cultural traits is not genetically inherited but learned, which means that any hypotheses based on the BIS need to take the broader sociocultural context into account to make a contribution to understanding attitudes toward immigrants and ethnic outgroups.

Empirically, the use of non-Western samples is a promising course to sensitize research on the BIS to the role of sociocultural context in the activation of psychological mechanisms. Along these lines, van Leeuwen and Petersen (2018) used samples from the US and India to test the impact of pathogen disgust sensitivity on attitudes toward ethnic outgroups. In a survey with the US sample, presented with photographs of Indians (represented by photographs of adult males with brown skin), they find that people with higher disgust sensitivity were less comfortable with contact with Indians. But they do not find such an association in the Indian sample when respondents were shown photographs of white males. They conclude that the association seems to be a result of processes that are culturally specific for the US or Western countries; importantly, they identify stereotypical beliefs about the trustworthiness and dirtiness of the non-white outgroup as responsible for the association. The possible role of stereotypes about hygienic conditions in an immigrant’s country of origin could be explored in a similar way: for example, in a survey administered to a sample from a stereotypically non-hygienic country that is asked about immigrants from a stereotypically hygienic country.

Theoretically, research on the BIS offers valuable insights into how and why stigmatization can be influenced by unconscious sensitivity: here, the focus is on disgust, but other unconscious processes, such as a need for physical safety, could play a role (Napier et al., 2018). Nevertheless, at present a significant part of the novelty appeal of evolutionary psychological approaches to explaining political attitudes and behavior relies on downplaying or omitting the role of social learning in the developmental unfolding and activation of psychological mechanisms. Talking about innate (Petersen and Kennair, 2009) or automatically operating (Aarøe et al., 2017) psychological mechanisms can be misinterpreted to mean an unchangeability of behaviors fixed in our evolutionary past (e.g., Kanazawa, 2001) that is not borne out by the empirical evidence (Varella et al., 2013). A careful consideration of the interplay between evolved psychological mechanisms and the changing and changeable social world in which these mechanisms affect behavior is not only the more fruitful course of research but also the more responsible one when it comes to the policy implications of such research. Understood in this way, research on the BIS could complement existing studies on a dual process model of prejudice and perceived group threat (Devine, 1989; Blinder and Lundgren, 2018) by offering a deeper understanding of automatic, unconscious reactions.

## Author Contributions

All authors listed have made a substantial, direct and intellectual contribution to the work, and approved it for publication.

### Conflict of Interest

The authors declare that the research was conducted in the absence of any commercial or financial relationships that could be construed as a potential conflict of interest.
